# Cognitive functional therapy versus therapeutic exercises for the treatment of individuals with chronic shoulder pain: A protocol for a randomized controlled trial

**DOI:** 10.1371/journal.pone.0320025

**Published:** 2025-04-21

**Authors:** Vander Gava, Jessica Mayara da Silva Eugenio, Ney Meziat-Filho, Lucas Araújo Almeida, Daniel Germano Maciel, Jose Diego Sales do Nascimento, Palloma Rodrigues de Andrade, Romário Nóbrega Santos Fonseca, Germanna Medeiros Barbosa, Valéria Mayaly Alves de Oliveira, Danilo Harudy Kamonseki

**Affiliations:** 1 Department of Physical Therapy, Universidade Federal de São Carlos, São Carlos, São Paulo, Brazil; 2 Postgraduate Program in Physical Therapy, Universidade Federal da Paraíba, João Pessoa, Paraíba, Brazil; 3 Postgraduate Program in Rehabilitation Sciences, Centro Universitário Augusto Motta, Rio de Janeiro, Rio de Janeiro, Brazil; 4 School of Rehabilitation Science, Faculty of Health Sciences, Institute for Applied Health Sciences, McMaster University, Hamilton, Ontario, Canada; 5 Department of Physical Therapy, University of Florida, Gainesville, Florida, United States of America; 6 Department of Physical Therapy, Universidade Federal da Paraíba, João Pessoa, Paraíba, Brazil; 7 School of Health Sciences of Trairi, Universidade Federal do Rio Grande do Norte, Santa Cruz, RN, Brazil; 8 Postgraduate Program in Physical Therapy, School of Health Sciences of Trairi, Universidade Federal do Rio Grande do Norte, Santa Cruz, RN, Brazil; Universiti Malaya, MALAYSIA

## Abstract

**Introduction:**

Shoulder pain is a debilitating musculoskeletal condition with functional, physical, and psychological impacts. Interventions for chronic shoulder pain should address the biopsychosocial model, with Cognitive Functional Therapy (CFT) emerging as a promising physiotherapy approach. CFT approaches the multidimensional nature of pain, integrating physical and cognitive aspects. To date, no study has assessed the effectiveness of CFT in individuals with chronic shoulder pain. Therefore, this randomized controlled trial aims to compare the effects of CFT to therapeutic exercises on pain intensity, disability, self-efficacy, sleep quality, biopsychosocial aspects, and central pain processing in individuals with chronic shoulder pain.

**Methods:**

This will be a randomized controlled trial, single-blinded with two parallel groups. Seventy-two individuals with chronic shoulder pain will be randomly assigned to one of two groups: CFT or Therapeutic exercise. The interventions will last 8 weeks, with the CFT group receiving therapy once a week and the therapeutic exercise group receiving sessions twice a week. The primary outcomes will be pain intensity and disability, while the secondary outcomes will include function, self-efficacy, sleep quality, biopsychosocial factors, perception of improvement/deterioration, and central pain processing. The outcome measures will be assessed at baseline, 4^th^ week, end of treatment (8^th^ week), and 12^th^-week follow-up.

**Conclusion:**

The results of this study will contribute to understanding the effectiveness of CFT in treating individuals with chronic shoulder pain.

**Trial registration number:** NCT06542666

## Introduction

Chronic shoulder pain has an estimated annual prevalence ranging from 0.7% to 55.2% in the general population [1]. It affects 45% of individuals engaged in repetitive work-related movements [2], pointing to an annual incidence of 37.8 per thousand people. Shoulder pain has significant physical, social, and psychological impact, often contributing to work limitations and difficulties in daily activities. This results in a significant economic burden on society due to the use of medication, sick leave, medical, and hospital expenses [3]. Furthermore, approximately 50% of individuals with shoulder pain continue to experience persistent pain after one year [4], indicating a high likelihood of chronification.

The condition is also linked to functional impairment and psycho-emotional distress, including depression, anxiety, decreased quality of life, kinesiophobia, catastrophizing, and low self-efficacy [3,5,6]. Thus, chronic shoulder pain presents multifactorial characteristics connecting the painful experience to fear-avoidance behaviors, which can further reduce function [6,7]. Therefore, interventions for individuals with chronic shoulder pain should align with the biopsychosocial model [8,9].

Therapeutic exercises are the primary recommendation for the treatment of shoulder pain [10–12]. These exercises typically focus on joint mobility, muscle stretching, and strengthening, often targeting the scapulothoracic and glenohumeral muscles [10,13,14]. However, given the strong association between psychosocial factors and the prognosis of chronic shoulder pain [5,15], exercise therapy alone may not adequately address the multidimensional nature of pain, as it fails to target the psychosocial aspects. This emphasizes the need for interventions that encompass physical, emotional, and behavioral components to yield more relevant and effective outcomes [16].

Cognitive Functional Therapy (CFT) is a patient-centered, individualized approach to address physical, psychological, emotional, and lifestyle barriers to improve pain symptoms, disability, and maladaptive cognitive-behavioral factors [17,18]. The CFT is structured into three phases: (1) making sense of pain, (2) exposure with control, and (3) lifestyle changes [19–21], including an approach for improving physical activity and sleep quality. Sleep has an important role in modulating physical and psychological health. Disruptions to sleep have been shown to significantly diminish quality of life and exacerbate the experience of pain in individuals with musculoskeletal conditions [22,23]. By contrast, physical activity has been identified as a key factor in promoting health, diminishing pain, and improving sleep quality, thereby influencing recovery and pain management outcomes [22,23]. Previous studies have demonstrated the positive effects of CFT on pain intensity, disability, and psychological factors in individuals with chronic low back pain [20,24–27], neck pain [28,29], and knee osteoarthritis [30]. Although CFT has shown significant and positive results for the treatment of chronic pain in other musculoskeletal conditions, randomized controlled trials investigating the application of CFT to treat chronic shoulder pain have not yet been conducted.

Therefore, this study aims to compare the effects of CFT with therapeutic exercise on biological aspects of pain (pain intensity, disability, function, perception of improvement/deterioration, and central pain processing), and psychosocial aspects of pain (sleep quality, self-efficacy, and biopsychosocial factors). We hypothesize that CFT will lead to greater improvements in these outcomes compared to therapeutic exercise.

## Methods

### Study design

This study is a randomized controlled trial with a superiority design, single-blinded with two parallel groups. Outcome assessors will be blinded to participants’ assigned treatment groups. This protocol follows the Standard Protocol Items: Recommendations for Interventional Trials (SPIRIT) statement and checklist [31]. This study was prospectively registered at clinicaltrials.gov (NCT06542666) and The World Health Organization data set is described in **Table 1**. The authors confirm that all ongoing and related trials for this intervention are registered.

**Table 1:** World Health Organization trial registration data set.

Data category	Information
Primary registry and trial identifying number	NCT06661681
Date of registration in primary registry	23/07/2024
Primary sponsor	Federal University of Paraíba
Contact for public queries	valeria.mayaly@gmail.com
Contact for scientific queries	Valéria Mayaly Alves de Oliveira,
Public title	Cognitive Functional Therapy for Chronic Shoulder Pain
Scientific title	Cognitive Functional Therapy versus therapeutic exercises for the treatment of individuals with chronic shoulder pain: A protocol for a randomized controlled trial
Countries of recruitment	Brazil
Health condition(s) or problem(s) studied	Chronic Shoulder Pain; Exercise Therapy; Psychosocial Rehabilitation
Intervention(s)	Cognitive Functional Therapy and Therapeutic Exercises
Key inclusion and exclusion criteria	Inclusion Criteria: men and women; age between 18 and 60 years; presence of shoulder pain for more than 3 months; pain intensity of 4 points or more on the 11-point Numeric Pain Rating Scale during the past week; high level of disability or moderate irritability. Exclusion Criteria: history of fracture or surgery of the clavicle, scapula, and/or humerus, surgical stabilization or rotator cuff repair; history of dislocation, instability (positive sulcus sign or apprehension test), and/or rotator cuff tear (positive drop arm test); adhesive capsulitis verified by the presence of gradual onset pain associated with stiffness and reduced passive and active mobility; ongoing pregnancy; reproduction of shoulder pain radiating to the entire upper limb during cervical or thoracic spine tests (positive Spurling test); neurological or systemic diseases that may alter muscle strength and sensitivity such as rheumatoid arthritis, fibromyalgia, lupus, gout, and diabetes; corticosteroid injection in the shoulder region in the last 3 months; physiotherapy treatment in the last 6 months.
Study type	Interventional
Date of first enrollment	February 14, 2025
Target sample size	72
Recruitment status	Not yet recruiting
Primary outcome(s)	Pain intensity and disability
Key secondary outcomes	Self-efficacy, Sleep quality, Biopsychosocial aspects, Central pain processing

### Research site

The evaluation and intervention procedures of this study will be conducted at the Laboratory for the Study of Balance, Dynamometry, and Electromyography and at the Physiotherapy School Clinic of the Universidade Federal da Paraíba, Brazil, in 2025.

### Sample

The study will include adults aged between 18 and 60 who report shoulder pain lasting at least 3 months. They should rate their shoulder pain 4 or higher on the 11-point Numerical Pain Rating Scale (NPRS) over the past week [32], and high level of disability (Shoulder Pain and Disability Index score ≥ 47) [33,34] or moderate irritability according to Staged Approach for Rehabilitation Classification: Shoulder Disorders (STAR–Shoulder) [35,36].

Non-inclusion criteria will be apply to individuals with: a history of fracture or surgery of the clavicle, scapula and/or humerus; surgical stabilization or repair of the rotator cuff; a history of dislocation, instability (positive Groove Sign or Seizure Test) and/or rotator cuff rupture (positive Arm Drop Test); adhesive capsulitis verified by the presence of pain with gradual onset associated with stiffness and reduced passive and active mobility; reproduction of pain in the shoulder that radiates to the entire upper limb, ongoing pregnancy; tingling or numbness in the upper limb or any other symptom in the upper limb during tests on the thoracic or cervical spine (Positive Spurling Test); systemic disease or neurological condition that can alter muscle strength and sensitivity, such as fibromyalgia, rheumatoid arthritis, gout, lupus, diabetes and/or stroke; physiotherapy treatment for shoulder pain in the previous 3 months, corticosteroid injection in the shoulder region in the last 3 months, and active treatment for cancer [37–39], impairments in sensation, blood clotting disorders, and/or contraindications to the application of ice.

Individuals with surgeries, fractures, musculoskeletal or neurological conditions preventing them from accessing treatment, or those who received corticosteroid injections in the shoulder region during the treatment or follow-up period will be discontinued from the study.

The sample size was calculated using the SampSize App (https://app.sampsize.org.uk/) and the following formula described by Flight et al (2015) [40]:


$$n=\frac{\left(r+1\right){\left(Z_{1-\beta }+Z_{1-\frac{\alpha }{2}}\right)}^{2}\sigma^{2}}{rd^{2}}$$

The calculation was based on an estimated mean difference of 18 points [41] (standard deviation of 24.6) [42] in shoulder disability, assessed with Shoulder Pain and Disability Index (SPADI), with a two-tailed significance set at 0.05, power at 80%, and accounting for a 15% dropout, which resulted in 36 individuals per group for a total of 72 individuals. The sample size, calculated based on pain intensity (a mean difference of 2 points and a standard deviation of 2.5 on the NPRS), resulted in a total sample of 48 individuals. Therefore, the sample size calculation for this study was based on shoulder disability.

### Recruitment

The recruitment of the individuals will be carried out between February 2025 and June 2026. Participants will be recruited through flyers posted in university buildings, orthopedic and rheumatology clinics, public places, and community health clinics in João Pessoa. Recruitment strategies will also include online advertisements (e.g., social media) and the personal and professional networks of the researchers involved in the study.

### Randomization

**Fig 1:**
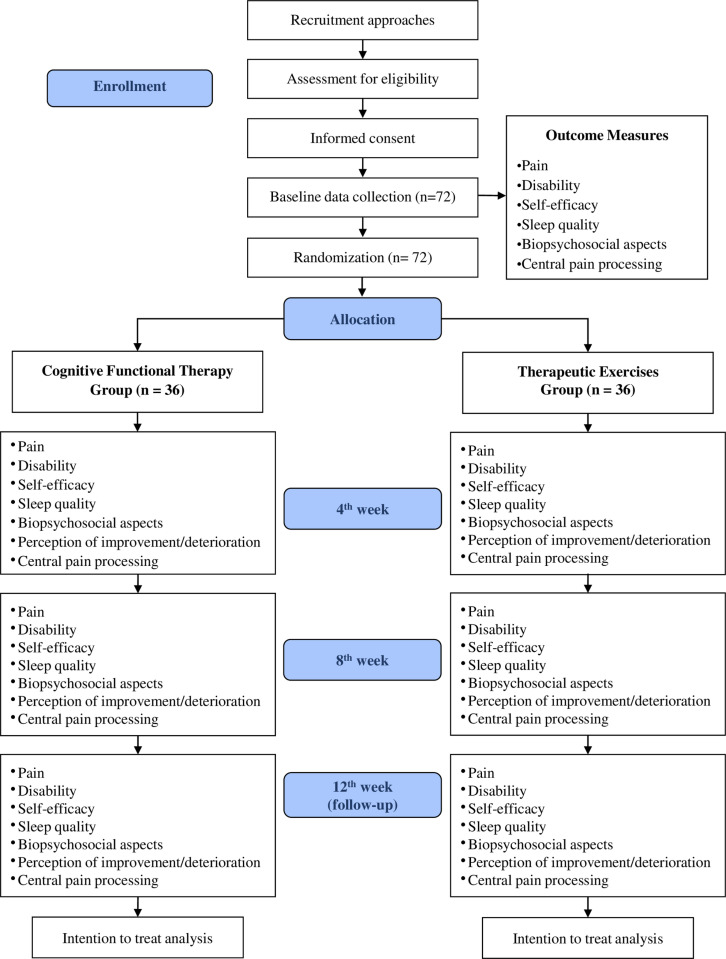
Diagram of participant flow.

Participants will be randomly assigned (1:1 ratio) to the CFT or Therapeutic Exercises groups (Fig 1). An independent researcher will carry out the randomization using a computer-generated sequence from http://www.randomization.com, stratified by age (<or ≥ 50 years) and sex (female or male), with group assignments concealed in consecutively numbered, sealed, opaque envelopes. These envelopes will be securely stored and opened sequentially by an independent researcher before the first session, ensuring allocation concealment.

### Blinding

The assessor and statistician will be blinded to treatment group assignments. Participants will be treated individually and not be informed of the study hypothesis. No circumstances have been established that would allow unblinding.

### Procedures

**Fig 2:**
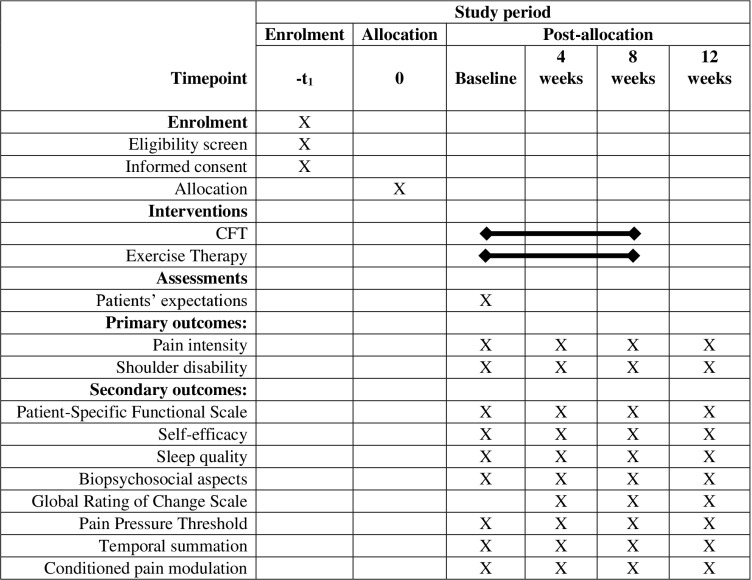
Schedule of enrolment, interventions, and assessments.

Baseline assessments will include the characteristics of the participants, such as sociodemographic data (e.g., sex of birth, age, height, weight, body mass index, occupation, education level, upper limb dominance, physical activity characteristics) as well as information related to shoulder pain (e.g., painful side, duration of symptoms, pain intensity, use of medication, influence of pain on work, leisure activities, and sleep, and details of previous treatments). The self-reported most painful shoulder will be considered for all outcome measures for individuals with bilateral symptoms. Outcome measures will be assessed at baseline, 4-week, at the end of treatment (8-week), and 12-week follow-up, except patients’ expectations of treatment, which will be measured only at baseline, and perception of improvement/deterioration, which will be measured at 4-week, 8-week, and 12-week follow-ups (Fig 2). Patients’ treatment expectations will be assessed using the question “How much do you expect your shoulder to improve at the end of treatment?”. The score will be measured on a 7-point Likert scale, where 1 corresponds to “the worst possible” and 7 corresponds to “fully recovered”. This assessment will be conducted before the intervention begins. Patient expectations are a significant predictor of treatment outcomes, as demonstrated by a study evaluating individuals with shoulder pain [43].

### Outcomes measures

The outcomes and outcome measures are presented in Table 2.

**Table 2:** Summary of outcome measures.

Outcome Measure	Type of Data	Scale	Unit of Measurement
**Primary Outcomes**			
Numerical Pain Rating Scale (NPRS)	Continuous	Ordinal (0–10)	Points
Shoulder Pain and Disability Index (SPADI)	Continuous	Interval (0–100)	Percentage (%)
**Secondary Outcomes**			
Patient-Specific Functional Scale (PSFS)	Continuous	Interval (0–10)	Points
Pain Self-Efficacy Questionnaire (PSEQ)	Continuous	Interval (0–60)	Points
Pittsburgh Sleep Quality Index (PSQI)	Continuous	Interval (0–21)	Points
Brief Screening Questions for Biopsychosocial Aspects	Continuous	Interval (0–10 per domain)	Points per domain
Global Rating of Change Scale	Ordinal	Ordinal (-7 to +7)	Points
Central Pain Processing Measures:			
Pressure Pain Threshold (PPT)	Continuous	Interval (kPa)	Pressure
Temporal Summation	Continuous	Interval (NPRS 0–10)	Points
Conditioned Pain Modulation (CPM)	Continuous	Interval (change in PPT)	Pressure

### Primary outcomes

#### Numerical Pain Rating Scale (NPRS).

The 11-point NPRS will measure shoulder pain intensity during functional activity involving arm elevation and the highest pain intensity experienced in the past week. The scale ranges from 0 (no pain) to 10 (maximum pain) and is a valid and reliable tool for assessing individuals with shoulder pain (intraclass correlation coefficient - ICC of 0.84) [42,44]. The minimum clinically significant difference (MCID) is a change of 2 points or 30% [45].

#### Shoulder Pain and Disability Index (SPADI).

The Brazilian version of the SPADI will be used to assess disability, composed of 13 questions related to two domains: disability (eight items) and pain (five items). The final score ranges from 0 to 100, with higher scores indicating greater shoulder disability [42,46]. The minimum detectable change (MDC) is 20 points or 43% of the baseline score [47].

### Secondary outcomes

#### Patient-Specific Functional Scale (PSFS).

The Brazilian version of the PSFS will be used to measure shoulder functional ability to complete specific activities. The individuals will select three to five important activities that are challenging to perform due to shoulder pain. For each reported activity, the individual will score on an 11-point scale, where 0 is (unable to perform the activity) and 10 is (able to perform the activity at the level before the injury) [48]. This scale is reliable, with an ICC of 0.91 and MDC of 1.5 points for patients with upper limb injuries [48]. Combining the scores of both SPADI and PSFS to assess individuals with shoulder pain will provide more insights into changes after treatment. The SPADI allows comparability between patients with preset questions, while the PSFS monitors changes in the most important activities impairing the function of each individual.

#### Pain Self-Efficacy Questionnaire (PSEQ).

The Brazilian version of PSEQ will be used to assess self-efficacy [49,50], which is composed of 10 items. The total score ranges from 0 to 60 points, with the higher score indicating greater self-efficacy. The scale is valid and reliable, with an ICC of 0.82 for assessing patients with chronic pain [51].

#### Pittsburgh Sleep Quality Index (PSQI).

The Brazilian version of PSQI will be used to assess sleep quality, which is composed of 19 items, resulting in a total score ranging from 0 to 21, and covering 7 domains: (1) subjective sleep quality, (2) sleep latency, (3) sleep duration, (4) habitual sleep efficiency, (5) sleep disturbance, (6) medication use and (7) dysfunction during the day [52]. The Brazilian version of PSQI is a valid and reliable tool, with a reliability coefficient of 0.82 [53].

#### Brief screening questions for biopsychosocial aspects.

The Brazilian version of Brief Screening Questions for patients with chronic pain will be used to assess biopsychosocial aspects [54], which is composed of six questions covering five domains: anxiety, kinesiophobia, stress, catastrophizing, and depression. Each question is scored on a scale from 0 to 10, with higher scores reflecting more severe symptoms [54].

#### Global rating of change scale.

The Global Rating of Change Scale will measure the individual’s perception of improvement/deterioration over time. Individuals will rate the overall change in their shoulder condition from baseline evaluation. The score ranges from -7–7, with higher scores representing a greater perception of health improvement, negative and lower scores indicating a worsening perception of health, and a score of zero representing no change [55].

#### Central pain processing.

The central pain processing will be assessed with a quantitative sensory test protocol [56] based on the modified order of the tests suggested by Gröne *et al*. 2012. The following test order will be performed: pressure pain threshold, temporal summation, and conditioned pain modulation [57]. This test order was predetermined to avoid possible carry-over effects of Conditioned Pain Modulation (cold stimulus) on other measures. Individuals will be blindfolded during those tests. A 5-minute rest will be provided between each sensory test.

i) Pressure Pain Threshold (PPT) is the minimum pressure that triggers the first pain sensation. PPT will be measured by a digital pressure algometer (AlgoMed ®, Hӧrby, Sweden) with a 1 cm^2^ circular rubber probe tip and a pressure rate of approximately 40 kPa/s, following established protocols [56,9]. Participants will be seated comfortably and instructed to maintain trunk stability during the test. The PPT test will be performed at the acromion and muscle bellies of the deltoid muscle belly (near the inferolateral insertion) and upper trapezius (halfway between the C7 spinous process and acromion process) on the affected or most affected side. PPT of a distant site from the shoulder (tibialis anterior) will also be assessed to investigate central sensitization [9]. The side for the tibialis anterior assessment will be randomly determined using a computer-generated randomization list (randomization.com). The non-painful side will be used as the remote site for participants with lower limb pain. A computer-generated randomization list will also determine the order of testing regions, stored in sealed opaque envelopes to be opened immediately before testing begins. Each site will be tested three times, with a 30-second interval between repetitions, and the average will be considered for analysis. Participants will undergo familiarization with the procedure at the lateral epicondyle. The PPT has been considered reliable, with ICC coefficients ranging from 0.82 to 0.97[58].
ii) Temporal summation refers to increased perceived pain from repetitive nociceptive stimuli at the same site, which is related to the modulation mechanism occurring in second-order sensory neurons in the spinal cord [59,60]. The temporal summation will be measured at the deltoid and tibialis anterior in the same position used for the PPT testing, with a 2-minute interval between testing regions. Sequential stimulation will involve 10 pressure stimuli at the pre-established PPT level, with a one-second interval between stimuli [9,61]. Each pressure increment will be approximately 40 kPa/s [9,61]. After the first and tenth stimuli, participants will rate their pain intensity using the NPRS. The temporal summation will be quantified by subtracting the pain intensity rating for the first stimulus from the pain rating for the tenth [9,61].
iii) Conditioned Pain Modulation (CPM) refers to the central nervous system’s ability to inhibit pain through mechanisms related to endogenous descending inhibition [9,62]. CPM will be assessed using the cold pressor test as the conditioning stimulus and PPT as the test stimulus. Cold pressor pain induces an effective CPM response when combined with the test stimulus of the PPT [63,64]. CPM will be evaluated in the same position used for the PPT. The participant will immerse their hand up to the wrist of the non-painful (or least painful) side [9] in ambient temperature water (22ºC) for 1 minute to standardize hand temperature [65]. Afterward, the individual will immerse the same hand in a cold-water bath (8°C) for 2 minutes. PPT at the deltoid of the affected (or most affected) side will be assessed at baseline, during immersion (after 30, 60, and 90 seconds), and after immersion (immediately post-withdrawal and at 30 and 60 seconds after withdrawal). Participants will rate their pain intensity on an 11-point NPRS every 30 seconds from immersion until hand withdrawal, and 30 and 60 seconds after withdrawal [66]. Conditioned pain modulation will be calculated by subtracting the PPT of the deltoid from PPT measures during and after CPM [62,64].

### Interventions

The interventions will last for 8 weeks, as individuals treated during this time frame have shown statistically and clinically significant improvements in previous research involving CFT [17,27] or therapeutic exercises for shoulder pain [12,13,67]. The CFT group will have one session per week, while the therapeutic exercise group will have two sessions per week. All treatment sessions will be supervised by a physiotherapist with 15 years of experience. Adherence to treatment sessions and assessments will be encouraged verbally at each session and through text messages sent the day before the scheduled appointment. In the event of absence, contact will be made by telephone to identify the reason for the absence and reschedule the session. Each session for both groups will last approximately 40–60 minutes.

Information regarding adverse effects, use of ice, heat, medication for pain, or protocol deviations reported by participants will be collected at all appointments by the physiotherapist responsible for administering the treatment and documented in the study report. An individual’s allocated intervention will be discontinued if they experience significant adverse effects related to the intervention or if they request to withdraw. The decision to discontinue the intervention will be made by the research team in a shared decision with the individual.

### Cognitive functional therapy

CFT will be conducted once a week in individual sessions lasting approximately 60 minutes over 8 weeks. The procedures of each session will be documented by the therapists for every participant. The therapists administering CFT will undergo specific training that includes: (1) a minimum of 106 hours of CFT training with an experienced CFT tutor, and (2) analysis of videos of the therapists implementing the CFT approach by an experienced physiotherapist. CFT is structured around creating a strong therapeutic connection, using active listening alongside a motivational interviewing style that is open, reflective, and non-judgmental. This approach provides validation and employs specific strategies to reinforce the therapeutic bond between therapist and patient [68,20]. The CFT will contain:

i) Anamnesis will be conducted to evaluate the degree of pain focus, pain coping strategies, stress response, and its connection to pain their beliefs regarding pain, as well as any history of anxiety and depression, and their expectations and goals regarding the management of the shoulder pain [69].
ii) Physical examination will be conducted to identify the individual’s primary maladaptive behaviors (e.g., pain-provoking, feared and/or avoided movements and/or functional tasks), that can be classified into movement impairments and control impairment. Movement impairments are characterized by movement restriction in the direction of pain and control impairments are impairments of functional control of postures and movements that can be associated with aberrant movement and postural control or muscle activity [26,70,71]. Subsequently, an approach focused on the potentially contributing factors to pain will be performed, covering the three following components [19,26]:
i) Making sense of pain is a reflective process that incorporates a patient’s story and relevant life experiences, alongside their behavioral exposure outcomes. This process helps them reach a new understanding of their pain and also increases self-efficacy to accomplish personal goals.

Developing a timeline of the pain history, along with recalling biopsychosocial factors that may have contributed to the onset and persistence of symptoms, can help make sense of their chronic pain and disability. Listening to the patient’s history is important to understand the context, location, and nature of their pain, assess their level of disability, beliefs, coping mechanisms, physical and lifestyle factors, as well as their goals and values.

The therapeutic alliance is strengthened through empathy, mirroring, reflective listening, and reinforcement of adaptive behaviors. Patients become aware of circumstances that lead to pain flare-ups, helping them recognize and explore coping strategies. Afterward, a summary of the discussion is reviewed with the patient.

ii) Exposure with control is a process of behavior change. This approach allows individuals to gradually resume meaningful activities without increasing pain or distress. It encourages viewing the pain experience as a hypothesis to be tested through behavioral experiments, making use of the pain experience whenever feasible. For instance, “raising my arm will increase my pain.” This is an experience where learned associations between threatening tasks and increased pain or harm can be corrected, and new “safety” associations are created. This strategy is based on the assumption that expectancy violation – the discrepancy between expectation and experience – is helpful for new learning.

Some individuals focus on reducing pain during task performance, while others prioritize engaging in feared or avoided tasks without causing harm. In this approach, sympathetic responses and safety-seeking behaviors that arise during painful, feared, or avoided tasks are specifically managed to create a contrast between the expected and actual pain experience. An example of prior individual expectation could be: “I expect my pain will increase if I raise my arm repeatedly.” Individual experience: “When I relax, breathe, and raise my arm without protecting it, my pain does not get worse – it improves.”

The practice of exposure with control improves relaxation before engaging in activities, reducing protective behaviors, and increasing body awareness and control. This approach helps individuals perform daily tasks more naturally. For example, calmly lifting the arm and adjusting movements to avoid unnecessary guarding can lead to a constructive experience through safety learning.

iii)  Lifestyle change aims to help patients embrace a healthy lifestyle. Strategies for altering unfavorable lifestyle behaviors are discussed as part of making sense of pain. Physical activity is tailored to individual preferences and aligned with personal goals, taking into account factors such as cost, accessibility, and social involvement to encourage lasting behavior change. Sleep deficits related to poor sleep hygiene are managed by creating a consistent daily routine and minimizing electronic device use in bed. Pain, anxiety, and stress-related disturbances can be managed with relaxation techniques like guided mindfulness and consistent physical activity. When difficulties with bed movement and positioning are identified, specific training is offered to promote more relaxed and comfortable rolling and positioning techniques [19,21,26].

### Therapeutic exercises group

Individuals assigned to this group will be treated twice a week for 8 weeks. Each session will last approximately 40–60 minutes and will consist of bilateral stretching and strengthening exercises targeting the scapulothoracic and glenohumeral muscles, which are commonly associated with shoulder pain [10,11,72]. In addition, the therapist will manage symptoms by applying ischemic compression for 90 seconds, or until symptom reduction, on latent or active trigger points in the deltoid and upper trapezius, as needed. Stretching exercises will be performed in three sets of 30 seconds, with 10 seconds of rest between sets, and an intensity of maximum stretch without pain, targeting the following muscles:

i) Upper trapezius: The individuals will be instructed to flex the neck, side-bend away, and rotate towards the side to be stretched, then force down the arm on the side being stretched. This movement can be assisted and maintained during the maneuver by the contralateral hand [38,39,73].
ii) Pectoralis minor: Individuals will be positioned with 90º of shoulder abduction, 90^o^ of elbow flexion, and forearm on the corner of a wall. Then, the individuals will shift the trunk forward and rotate opposite to the side being stretched [74].
iii) Posterior shoulder region: the individuals will be positioned in supine and the therapist will stabilize the scapula placing one hand on the lateral edge of the scapula, and with the other hand, the therapist will perform shoulder horizontal adduction [75].

Strengthening exercises will be performed using dumbbell or elastic resistance bands (Theraband ®) with 4 progressive levels of resistance: red (medium), green (heavy), blue (extra heavy), and gray (super heavy). The starting level of resistance will be adjusted for each individual based on their strength and ease of performing the exercise correctly. The individuals will perform three sets of 10 repetitions, with one minute rest between sets, for each strengthening exercise. The progression of resistance will be regulated individually using the 11-point rating of perceived exertion (RPE) scale according to the corresponding muscle being exercised [76], validated for quantifying the intensity of resistance exercise [77]. The scale values range from 0 (“no effort”) to 10 (“maximum effort”) and participants will be instructed in advance on how to report their exertion using the scale and will report after every set of exercises [78]. If self-reported pain occurs during exercise, the exercise will continue as long as the pain does not worsen or hinder proper execution. Otherwise, the therapist will reduce the resistance. The individuals will perform the exercises at a moderate intensity (PSE score between 3–5 points) and the resistance will be adjusted by changing the length or the elastic resistance. The strengthening exercises will be as follows [10,38,39,73]:

i) Lower trapezius - Prone extension exercise: The individual will be positioned in the prone position, with the shoulder in 90º flexion and neutral rotation, and extended elbows. Afterward, the individuals will perform the shoulder extension movement to the neutral position (arm at the side of the body) holding a dumbbell [79,80].
ii) Middle trapezius - Horizontal abduction exercise with lateral rotation: The individual will be positioned in the prone position, with the shoulder at 90º flexion and external rotation, and extended elbows. Then, the individuals will perform the horizontal abduction movement holding a dumbbell [80]
iii) Serratus punch exercise: The individual will be positioned in the supine position, with the shoulder at 90º flexion, elbows extended and will perform the shoulder protraction holding a dumbbell [81].
iv) Shoulder external rotators: The individual will be positioned standing, with the elbow at 90° flexion and shoulder adducted and internally rotated. Then, the individuals will be instructed to perform external rotation against elastic resistance [80,82].
v) Shoulder internal rotators: The individuals will be positioned standing, with elbow at 90° flexion and shoulder adducted and laterally rotated. Afterward, the individuals will be instructed to perform the internal rotation against elastic resistance [80,82].

### Data management

Information regarding recruitment, participant characteristics, study completion, dropout rates, and outcome measures will be securely stored at the Universidade Federal da Paraíba. To protect individuals’ privacy and data, identifiers will be stored separately. Data will be entered into a computer software program (Excel™, Microsoft, 2016) and cross-checked on a weekly basis by an assistant, using standardized coding to maintain participant confidentiality. Access to the database will be restricted to the primary investigators. There are no contractual agreements limiting investigator access.

### Data analysis

Statistical analysis will be conducted using the Statistical Package for the Social Sciences (SPSS) version 24.0 or a more recent version (SPSS Inc., Chicago, IL). Continuous data will be summarized as means ± standard deviation (SD) and 95% confidence intervals (CIs). Data normality will be assessed through visual inspection of histograms and the Shapiro-Wilk test. The analysis will adhere to the intention-to-treat principle. Between-group differences (treatment effects) and their corresponding 95% CIs will be computed using linear mixed models with Bonferroni adjustments. If non-normality of the data is identified, transformations will be performed as needed or generalized linear mixed models will be considered. Fixed effects will include group, time, and the group-by-time interaction. Random effects will account for individual variability. A significance level of 0.05 will be applied for all statistical tests.

### Data monitoring

Monitoring will be conducted by a researcher independent of the trial investigators, to ensure data integrity and trial compliance. No interim analyses or stopping are planned for this study. The analysis will be conducted after the completion of all recruitment and data collection.

### Harms

Adverse events and unintended effects will be collected and reported throughout the trial and to the Human Research Ethics Committee of the Universidade Federal da Paraíba. Individuals will be asked to report any side effects, which will be analyzed by the research team. All adverse events will be evaluated by their severity and potential relation to the interventions. The use of pain relief methods, such as medication, ice, or heat packs, during the study period will also be recorded.

### Auditing

An independent researcher will review the study’s progress, audit the quality and completeness of the data every six months, and ensure that all protocol procedures are being implemented as planned.

## Ethics and dissemination

### Research ethics approval

This trial protocol was approved by the Research Ethics Committee at Universidade Federal da Paraíba (CAAE: 84462524.8.0000.5188). Any changes made to the research protocol will be submitted to the Research Ethics Committee and registered on the clinical trial registration platform.

### Protocol amendments

Any protocol amendments will be previously requested to the ethical committee and then informed to all relevant parties, including trial registries and participants.

### Ancillary and post-trial care

No provisions are planned for ancillary or post-trial care. Compensation for any harm related to trial participation will be provided per institutional guidelines.

### Dissemination policy

The trial results will be disseminated through peer-reviewed publications, conference presentations, and public reports. Researchers who make substantial contributions to the study’s design, execution, interpretation, or reporting will be credited as authors in the final publication, and no professional writers will be involved in the manuscript preparation. Access to the full protocol, participant-level dataset, or statistical code will be available upon request.

## Discussion

Rehabilitation for shoulder pain can be prolonged and place a burden on healthcare systems, highlighting the need for more effective treatment solutions. Pain is influenced by biological, psychological, emotional, and social factors, yet standard treatments often overlook psychosocial aspects. CFT is a recent and promising approach for treating various musculoskeletal disorders, but its effectiveness in shoulder pain has not yet been explored. The results of this randomized controlled trial could provide valuable evidence supporting the use of CFT to enhance shoulder pain rehabilitation.

### Strengths and limitations

This RCT will provide evidence of the use of CFT in individuals with shoulder pain. The methods follow established guidelines for conducting and reporting trials, with an appropriate sample size calculation to provide statistical power in all outcomes, blinded assessor, and stratified randomization process. The authors have significant experience in developing and delivering interventions involving CFT and conducting RCTs with patients with shoulder pain. Therapists providing CFT will receive appropriate training and supervision to ensure proper delivery of the intervention [83]. The comparator group, therapeutic exercises, follows current best practices, offering a fair comparison to CFT. Additionally, the study focuses on patients with high levels of disability, a population that typically reports lower levels of improvement after rehabilitation [43], adding further relevance to our findings. Therefore, we believe this study will contribute to the best practice evidence for treating patients with shoulder pain.

A key limitation is the inability to blind therapists and participants due to the nature of the intervention. Both will need to be aware of the study’s objectives and treatments. Finally, another limitation is that CFT, like therapeutic exercise, lacks established parameters and dosage, as the research on CFT is still recent.

## Supporting information

S1 File SPIRIT Checklist. (PDF)
